# Sertraline-Induced Bradycardia: A Case Report and Literature Review

**DOI:** 10.7759/cureus.84474

**Published:** 2025-05-20

**Authors:** Narges Joshaghani, Jaskaran Singh, Ravleen K Suri, Daniel Udegbe, Ajiya Fatima, Sakshi Prasad, Khai Tran, Sasidhar Gunturu

**Affiliations:** 1 Psychiatry, BronxCare Health System, New York, USA; 2 Research, School of Medicine, DY Patil University, Delhi, IND; 3 Psychiatry, Godfrey Okoye University Teaching Hospital, Enugu, NGA; 4 Internal Medicine, Allied Hospital, Faisalabad, PAK

**Keywords:** antidepressants, bradycardia, major depressive disoder, sertraline, ssri

## Abstract

Selective serotonin reuptake inhibitors (SSRIs) are first-line treatments for depression and several psychiatric disorders due to their effectiveness and tolerability. However, they can cause cardiovascular side effects, particularly bradycardia, raising safety concerns. SSRI-induced bradycardia occurs through serotonin's effects on autonomic and cardiovascular regulation. SSRIs impact cardiac conduction by modulating serotonin receptors and inhibiting sodium and calcium channels, leading to varied bradycardia presentations. This case report indicates the occurrence of bradycardia in a 63-year-old male patient after an increasing dose of sertraline, an SSRI. The patient exhibited symptoms of bradycardia (heart rate of 46 bpm) along with headache and dizziness. The patient also had a medical history of hypothyroidism and hyperlipidemia. The pharmacological intervention involved gradually increasing the dose of sertraline from 25 mg to 50 mg daily to optimize the treatment of depression, while bradycardia persisted. A switch to escitalopram and dose adjustments of levothyroxine did not resolve the issue. Ultimately, the bradycardia resolved after switching to bupropion. This contrasts with previous reports where elderly patients experienced rapid resolution of bradycardia after discontinuation of SSRIs. Our patient exhibited bradycardia as opposed to severe outcomes reported in other studies, further showing the unique nature of this case. The study highlights the significant increase in mortality risk due to cardiovascular side effects associated with psychiatric medications. While SSRIs are generally regarded as safer compared to other antidepressants, previous literature reviews indicate a notable potential for cardiovascular side effects. The literature review examines various databases and reports that while citalopram, escitalopram, and fluoxetine have been linked to bradycardia at elevated doses, there is limited evidence directly connecting sertraline to this condition. This study concludes by emphasizing the need for careful monitoring and consideration of various factors, such as age, ethnicity, and the potential for polypharmacy, when prescribing SSRIs. The findings underscore the necessity for further research to elucidate the relationship between sertraline and bradycardia and inform clinical management strategies for this adverse reaction.

## Introduction

Managing psychiatric disorders can substantially raise the risk of mortality due to disabling cardiovascular side effects noted with psychiatric medications [1,2]. There has been a notable increase in the number of prescriptions having antidepressant medications over the last years [3]. Furthermore, a meta-analysis study in 2019 reported that the likelihood of experiencing cardiovascular side effects from antidepressants could be as high as 0.89% [4]. These antidepressants have been reported to negatively impact cardiovascular health and cause events like bradycardia, tachycardia, hypertension, hypotension, orthostatic hypotension, electrocardiogram (ECG) changes, electrolyte abnormalities, reduced cardiac conduction and output, arrhythmias, and sudden cardiac death [5].

An animal study examined the impact of various antidepressants on rat heart embryos in vitro and found that, at higher concentrations, most antidepressants caused sodium channel blockade in the heart muscle, while some induced negative inotropy by blocking L-type calcium channels [6]. This can further explain the medication’s cardiotoxic traits at a dose higher than the therapeutic dose [7]. Selective serotonin reuptake inhibitors (SSRIs), including citalopram, escitalopram, fluoxetine, fluvoxamine, sertraline, paroxetine, etc., are claimed to be safer and less toxic than the other classes. However, with the increasing number of case reports [8-22], the use of SSRIs has been reported to cause adverse complications like orthostatic hypotension, mild bradycardia, and QT interval prolongation [23,24]. This has ultimately led to the growing discussions regarding their safety profile [25].

We analyzed case reports available on databases, like PubMed, Scopus, ScienceDirect, and Google Scholar, that provided any anecdotal complications or unusual cardiac events with the use of antidepressants that were similar to our case presentation. We found that SSRIs like citalopram, escitalopram, and fluoxetine have been reported to cause mild reduction in heart rate and other cardiotoxic side effects at higher doses [9,15,26-34]. To the best of our knowledge, there is limited to no evidence in the literature that associates sertraline, an SSRI, with the onset of bradycardia. It is important to bridge this gap in our understanding as physicians, even though such adverse events related to this medication can be rare. Given the unclear relationship between this antidepressant use and the risk of bradycardia, we present a case of symptomatic bradycardia in a patient who had been taking 50 mg of sertraline daily. We have also discussed the patient's clinical course and management to emphasize the cardiotoxic side effects linked to sertraline use.

## Case presentation

The patient is a 63-year-old Asian man, married, currently unemployed, and living with his family. He has a psychiatric history of major depressive disorder (MDD) and a medical history of hypothyroidism and hyperlipidemia. 

The patient presented to the Comprehensive Psychiatric Emergency Program (CPEP) accompanied by his therapist from the outpatient clinic due to worsening depression and active suicidal ideation, including a plan to jump in front of traffic. He reported being deterred from acting on these thoughts by concerns for his family. 

The patient described a four-month history of progressively worsening depressive symptoms, including depressed mood, anxiety, loss of appetite, insomnia, anhedonia, hopelessness, social withdrawal, and suicidal ideations (frequent episodes lasting about an hour, occurring several times a day). He attributed his depression to ongoing financial issues and the stress of an associated legal trial, which he fears will negatively impact his family. 

The patient did not report prior inpatient psychiatric admissions or emergency room (ER) visits, which was consistent with findings in Psychiatric Services and Clinical Knowledge Enhancement System (PSYCKES). He also denied any history of suicide in the past or use of illicit substances or drinking alcohol, which was confirmed by a negative urine toxicology screen. The patient recently began outpatient psychiatric treatment for the first time two months prior, during which he was prescribed sertraline 50 mg daily and trazodone 50 mg at bedtime. He was also prescribed levothyroxine 100 mcg daily and atorvastatin 20 mg at the same time for hypothyroidism and hyperlipidemia. The patient reported compliance with the medications. 

Upon admission to CPEP, the patient was found to have an abnormal echocardiogram (ECG), sinus bradycardia (ventricular rate 39) with complaints of headache and dizziness. He was transferred to the medical ER for evaluation and pertinent workup. Further cardiac evaluation revealed no structural or functional abnormalities, and the ECG demonstrated normal left ventricular contractility with an ejection fraction of 72.98%. The pro-brain natriuretic peptide (BNP) level was slightly elevated, while the thyroid-stimulating hormone (TSH) and troponin levels were within normal limits (Table 1). The patient was deemed asymptomatic, the dose of sertraline was reduced to 25 mg, and the levothyroxine dose was reduced to 75 mcg daily due to bradycardia. The patient was medically cleared and transferred back to CPEP to continue treatment for his depression and suicidality.

**Table 1:** Pertinent labs TSH: thyroid-stimulating hormone; T3: triiodothyronine; BNP: brain natriuretic peptide

Pertinent labs	Values	Reference range
TSH	2.81	0.40-4.50 mIU/L
T3	53.1	60-181 ng/dL
Free thyroxine	1.12	0.80-2 ng/dL
Microsomal thyroid antibody	10.1	<35 IU/mL
Troponin	6	<12 ng/L
Pro-BNP	161	0-125 pg/mL
Procalcitonin	<0.02	0.02-100 ng/mL

**Figure 1:**
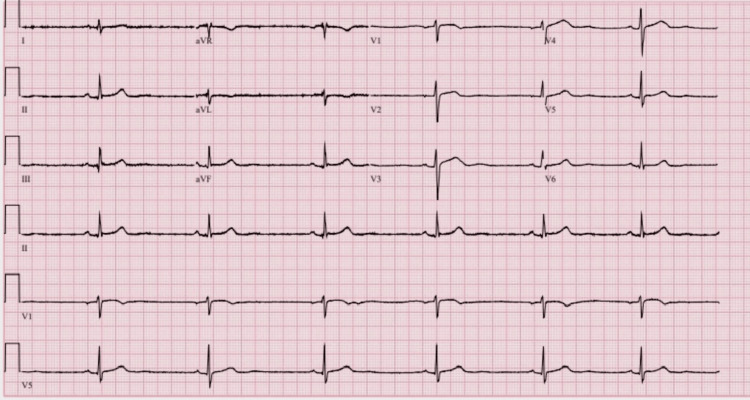
ECG image during treatment with Sertraline 50 mg daily

**Figure 2:**
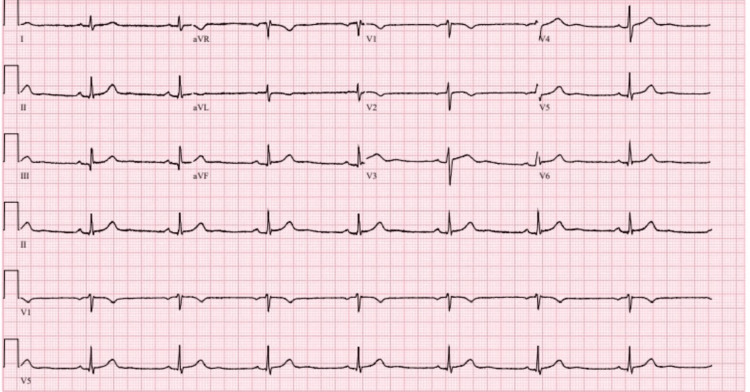
ECG image during treatment with sertraline 25 mg daily ( dose reduced from 50 mg to 25 mg daily) ECG: echocardiogram

**Figure 3:**
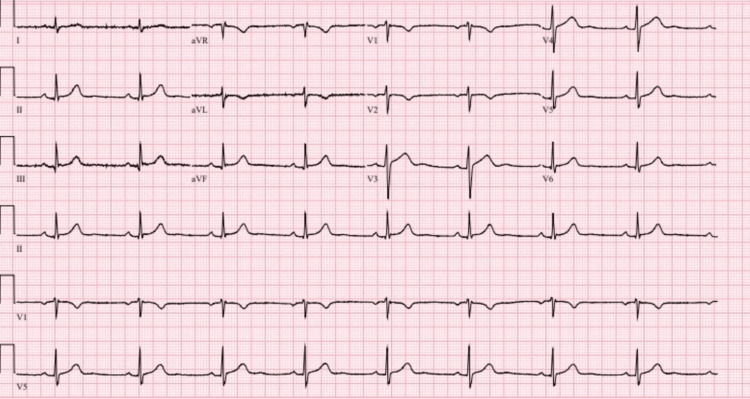
ECG image during treatment with escitalopram 10 mg daily ECG: echocardiogram

**Figure 4:**
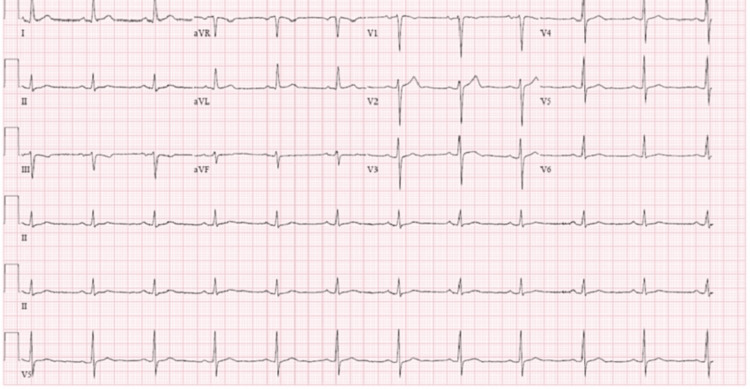
ECG image during treatment with bupropion XL 150 mg daily ECG: echocardiogram

The initial pharmacological intervention for the patient involved a continued daily dose of 25 mg of sertraline. Concurrently, the patient resumed taking levothyroxine at the dose of 75 mcg every morning. Nevertheless, daily clinical observations revealed the patient's heart rate ranging from 48 to 55 bpm (beats per minute). Serial ECGs after each dose change continued to demonstrate sinus bradycardia without other significant abnormalities (see Figures 1-4, Table 2).

**Table 2:** ECGs finding and pulse rates during treatment with different antidepressants ECG: electrocardiogram

ECGs	Findings	Pulse rates	Medication and dosage
Figure 1	Marked sinus bradycardia, otherwise normal ECG (ventricular rate 39 bpm); QTc 376 ms	42-53	Sertraline 50 mg daily (home med)
Figure 2	Sinus bradycardia, otherwise normal ECG (ventricular rate 46 bpm); QTc 400 ms	48-55	Sertraline 25 mg daily
Figure 3	Sinus bradycardia, otherwise normal ECG (ventricular rate 51 bpm); QTc 405 ms	51-62	Escitalopram 10 mg daily
Figure 4	Normal ECG (ventricular rate 68 bpm); QTc 398 ms	60-67	Bupropion XL 150 mg daily

Consultation with an internist specialist prompted a therapeutic adjustment, transitioning the patient from sertraline to escitalopram 10 mg daily and advancing the levothyroxine dosage from 75 mcg to 100 mcg daily for managing hypothyroidism. Subsequently, the patient consistently denied any suicidal intentions, plans, or attempts, while expressing an improved mood on escitalopram after a week of administration. Despite this improvement, the patient's pulse rate persisted within the range of 51-62 bpm throughout this dosage crossover. In response, there was a recommendation to escalate the levothyroxine dose to 112 mcg, although the bradycardia remained unresolved. With consideration for the recurrent decline in heart rate, the patient was transitioned off escitalopram and initiated on extended-release bupropion at a dosage of 150 mg daily. Following this adjustment, the heart rate reached a consistent range of 60-67 bpm and above over time. Ultimately, upon achieving psychiatric stability, the patient was discharged on bupropion 300 mg and levothyroxine 112 mcg, ensuring continued management of his mental health condition.

Comprehensive collaboration was established with the community service, involving virtual meetings with family and the outpatient mental health program to ensure continuity of care and communication regarding the patient's treatment progress and plans for discharge. The patient exhibited significant improvement in depressive symptoms. He began interacting socially with peers on the unit, attended group activities selectively, and demonstrated brighter affect and making intermittent eye contact and smiling during the interview, as well as engaging actively in the treatment plan. He no longer reported active suicidal thoughts, intent, or plans and reported an improved sleep pattern and appetite. The discharge plan aimed to facilitate the patient's compliance with medication, follow-up appointments at the outpatient clinic, and continued family support, showing a holistic approach to his mental health management, considering both medical and psychiatric aspects of his care.

## Discussion

Prevalence and mechanism of SSRI-induced bradycardia

SSRIs are commonly used as first-line pharmacological agents for the treatment of anxiety, depression, and other psychiatric disorders due to their better tolerability and efficacy [35]. However, regardless of their safety profile, there are many cardiovascular side effects, including tachycardia, bradycardia, hypertension, ECG changes, orthostatic hypotension, electrolyte abnormalities, reduced cardiac conduction and output, arrhythmias, and sudden cardiac death that have been documented, raising concerns regarding their cardiotoxic potential in various patients [5,24]. Among SSRIs, citalopram has been known to have the highest cardiotoxic capacity, especially in a dose-dependent manner [35,36]. For instance, another study revealed that citalopram-treated patients demonstrated reductions in heart rate within the first week of treatment, with 3-4% of patients developing bradycardia, even in those with normal baseline heart rates [36].

The basic mechanism of SSRI-induced bradycardia remains an area of keen investigation. Currently, the evidence indicates serotonin’s influence on autonomic and cardiovascular regulation portrays a central role, as SSRIs modulate serotonin receptors in ways that may modify cardiac conduction [37,38]. Experimental studies have further assisted this hypothesis, with findings that citalopram and fluoxetine inhibit L-type calcium channels and sodium channels in the heart, pertaining to slowed cardiac conduction and bradycardia in animal models [39,40]. These vasodilatory and cardiodepressant properties may account for the different presentations of bradycardia, from mild to life-threatening, across variable clinical cases. 

Comparing different case reports

When correlating our case of SSRI-induced bradycardia with past case reports (Table 3), several unique aspects emerged. Our patient originally presented with bradycardia after starting sertraline and continued to demonstrate a low heart rate despite transitioning to escitalopram, highlighting a consistent bradycardic response across various SSRIs. This is contrasting with regard to the cases observed by Ozdilek [18] and Isbister et al. [41], where elderly patients experienced swift resolution of bradycardia within 24-48 hours after cessation of escitalopram or citalopram, without the need for any further interventions. Similarly, Favre et al. in 1999 reported QTc normalization and resolution of bradycardia within 24 hours after discontinuation of citalopram, indicating that, in many cases, the adverse side effect resolves rapidly following drug discontinuation [11]. However, reports by Padala et al. and Brucculeri et al. revealed dose-dependent relationships, with repetition of bradycardia upon SSRI rechallenge or intentional overdose, deepening the importance of vigilant monitoring and careful dosing [19,10].

**Table 3:** Studies reporting SSRI-induced bradycardia SSRIs: selective serotonin reuptake inhibitors; ECG: electrocardiogram; IV: intravenous

Name of the study; year	Type of study	Dosage and mode of administration	Intervention	Outcome
Severe bradycardia following fluoxetine administration: a case report in a young HIV-1 infected woman; 1995 [13]	Case report (34-year-old female patient)	Fluoxetine 20 mg/day orally: severe sinus bradycardia after 12 days	Fluoxetine stopped, HR monitored, IV atropine for HR <30 bpm, cardiac monitoring, brain stem MRI, and ANS evaluation	HR normalized in ~25 days after fluoxetine discontinuation
Suicide attempt by pure citalopram overdose causing long-lasting severe sinus bradycardia, hypotension and syncopes: successful therapy with a temporary pacemaker; 2000 [21]	Case report (32-year-old female patient)	Citalopram, initially 20 mg/day orally, ingested 800 mg in a suicide attempt: sinus bradycardia within 4 hours	Gastric lavage, active charcoal treatment, cardiac and biochemical monitoring, IV atropine, and temporary pacemaker placement	Pacemaker removed on day 7 after sinus rhythm stabilized; discharged on day 11 in good physical health and admitted to the psychiatric hospital
Bradycardia during citalopram treatment: a case report; 1999 [11]	Case report (47-year-old female patient)	Citalopram, initially 20 mg/day orally, increased to 40 mg/day; atenolol 50 mg/day: sinus bradycardia and prolonged QTc after discontinuing atenolol while on citalopram 40mg/day	Citalopram was immediately discontinued, and ICU monitoring for potential arrhythmias	After stopping citalopram, HR gradually normalized, and QTc shortened to normal limits. Venlafaxine was later initiated at 150 mg/day, resulting in a good clinical response with no further bradycardia
Relative toxicity of selective serotonin reuptake inhibitors (SSRIs) in overdose; 2004 [15]	Case report (60-year-old female patient)	Citalopram 20 mg/day orally: symptomatic bradycardia and mild hypotension after 2 weeks	Citalopram was discontinued, and the patient's cardiac function was closely monitored	Symptoms resolved within 48 hours of discontinuing the drug, with no QTc prolongation noted
Severe bradycardia in a stroke patient caused by a single low dose of escitalopram; 2007 [9]	Case report (60-year-old female patient with ischemic stroke)	Escitalopram 5 mg/day orally started on the 3rd day post-stroke: sinus bradycardia and respiratory failure within 45 minutes	Immediate resuscitation, continuous monitoring in the stroke unit, with discontinuation of the drug	Resuscitation was successful, and bradycardia resolved; however, pre-existing cerebral hypoperfusion worsened, increasing the deficit. The patient had a previous adverse reaction to citalopram, suggesting possible hypersensitivity to SSRIs
Dose-dependent bradycardia with citalopram in an elderly patient; 2010 [19]	Case report (66-year-old male patient)	Citalopram: initially 10 mg/day orally. Later increased to 20 mg/ day: symptomatic sinus bradycardia	Citalopram dosage was reduced back to 10 mg/day	HR normalized and symptoms resolved upon reduction of dose. A rechallenge at 20 mg resulted in a prompt return of bradycardia, confirming a dose-dependent relationship
Reversal of citalopram-induced junctional bradycardia with intravenous sodium bicarbonate; 2005 [10]	Case report (82-year-old female patient)	Citalopram 1.6 g intentional oral overdose: prolonged QT/QTc intervals, sinus bradycardia, junctional escape rhythm, and tonic-clonic seizures	Sodium bicarbonate 50 mEq was administered intravenously twice, followed by a continuous infusion of 150 mEq in 1 L of 5% dextrose in water.	Sodium bicarbonate intervention temporarily improved the ECG findings, and after 36 hours of continuous infusion, the patient's heart rhythm returned to normal sinus with improved QT/QTc intervals.
Escitalopram-induced bradycardia in elderly individuals: a case series report; 2015 [18]	Case series (patient 1: 65M; patient 2: 70F; patient 3: 75F)	Pts 1 and 2; escitalopram 10 mg/day orally Pt 3; escitalopram 5 mg/day orally: sinus bradycardia in all 3 pts	Discontinuation of escitalopram in elderly patients 1, 2, and 3	Sinus bradycardia resolved within 24 hours after discontinuation of escitalopram in all 3 elderly patients. Pts 2 and 3 later treated with venlafaxine with good response
Adverse effects of interactions between antidepressants and medications used in treatment of cardiovascular disorders; 2019 [22]	Case analysis (66 cases with adverse cardiac reactions)	SSRIs combined with beta-blockers (metoprolol, propranolol); orally, the dosage varies	Cardiac functioning is monitored after SSRIs combined with beta-blockers for cardiovascular disorders	In 37.9% of cases (n = 25), bradycardia was observed as a side effect of combining SSRIs with beta blockers (metoprolol or propranolol)
Bradycardia caused by interaction of venlafaxine and cyclosporine: a case report; 2019 [8]	Case report (38-year-old female patient)	Cyclosporine 50 mg twice daily orally; single dose of venlafaxine 37.5 mg orally added: sinus bradycardia	Cardiac monitoring and discontinuation of venlafaxine and cyclosporine	Bradycardia resolved within 48 hours upon discontinuation of venlafaxine and cyclosporine, suggesting a drug interaction between the two
Metoprolol and sertraline combined treatment may increase the risk of bradycardia; 2010 [20]	Case report (64-year-old female patient)	Metoprolol 25 mg/day orally, sertraline 25 mg/day orally: symptomatic sinus bradycardia	​​Cardiac monitoring with discontinuation of metoprolol. Then metoprolol was replaced with bisoprolol 2.5mg/day. Continued use of sertraline was not specified	After 12 hours of discontinuation of metoprolol, the patient’s heart rate stabilized
Therapeutic single-dose mirtazapine-induced symptomatic bradycardia: a case report; 2018 [14]	Case report (48-year-old female patient)	Single dose of 30 mg of mirtazapine orally: symptomatic bradycardia within 30 minutes	Drug was discontinued with cardiac monitoring. Half a mg of intravenous atropine and a theophylline inhaler were administered	Bradycardia resolved within 36 hours of discontinuation. venlafaxine 75mg/day started. The patient’s complaints did not return in control examinations
Escitalopram-induced sinus bradycardia in coronary heart disease combined with depression: a case report and review of literature; 2024 [17]	Case report (82-year-old female patient with coronary heart disease)	Escitalopram 5 mg/day orally, increased to 15 mg/day; Lorazepam 0.5mg/day; Digoxin 0.125mg; Clopidogrel 75mg/day; Isosorbide mononitrate 40 mg/day; atorvastatin, furosemide, and spironolactone 20 mg/day; recurrent sinus bradycardia and sinus arrest episodes	Cardiac monitoring with discontinuation of escitalopram, lorazepam, and digoxin. Clopidogrel, spironolactone, atorvastatin, isosorbide mononitrate, and furosemide continued till 180-day course completion	Sinus bradycardia significantly decreased from 93.7% (before escitalopram discontinuation) to 0.1% (after escitalopram discontinuation). Escitalopram was restarted after discharge, but was later discontinued again after worsening symptoms and monitoring of the ECG

With regard to severity, our case presented with mild bradycardia that was asymptomatic apart from dizziness and headache, which contrasts with severe results observed in other cases. For instance, Rothenhäusler et al. [21] and Beyenburg et al. [9] displayed life-threatening complications, including syncope requiring temporary pacemaker insertion and resuscitation. Similarly, Grassi et al. described fluoxetine-induced bradycardia pressing atropine administration, with recovery that was delayed over a prolonged course of 25 days, in contrast to our patient’s steady improvement after transitioning to bupropion [13]. Interestingly, Gundogmus et al. linked mirtazapine-induced bradycardia to hypersensitivity, which resolved rapidly upon discontinuation, further underscoring the variability in clinical presentations and recovery timelines [14].

In our case, despite transitioning from sertraline to escitalopram and then to bupropion, bradycardia only resolved with the cessation of SSRIs altogether. This suggests an unusual sensitivity to serotonergic agents affecting heart rate. Thus, our case contributes to the existing literature by demonstrating that bradycardia can persist across different SSRIs and may necessitate complete discontinuation to achieve heart rate normalization. 

Influence of concurrent drugs on SSRI efficacy and safety

The interplay between SSRIs and other medications has yielded varied clinical outcomes. The role of polypharmacy turns out to be crucial in many reports. Woroń et al. [22] and Protopapas et al. [20] highlighted the exacerbation of bradycardia when SSRIs were combined with beta-blockers, such as metoprolol. This aligns with the pharmacogenomic findings of Thomas et al., who reported that patients homozygous for the Arg389/Arg389 ADRB1 SNP may experience amplified reductions in heart rate when prescribed beta-blocking SSRIs such as fluoxetine or paroxetine [42]. This is distinct from our case, where no cardiovascular medications were involved. Interestingly, Azizi et al. [8] reported bradycardia resulting from a drug interaction between cyclosporine and venlafaxine, and Li et al. [17] observed severe outcomes, including sinus arrest, when escitalopram was used alongside digoxin in an elderly patient with coronary artery disease. These findings contrast sharply with our patient, whose bradycardia persisted in a relatively straightforward pharmacological context, further highlighting the need for caution even in the absence of polypharmacy.

In contrast, interactions between SSRIs and levothyroxine appear to involve endocrine rather than cardiovascular dynamics. Benvenga, Eker et al., and Harel et al. noted that SSRIs like sertraline and escitalopram could suppress thyroid-stimulating hormone (TSH), necessitating increased doses of levothyroxine for effective hypothyroidism management [43-45]. This observation resonates with our case, where upward titration of levothyroxine accompanied the management of SSRI-induced bradycardia, hinting at a possible interplay between thyroid function and SSRI metabolism. Conversely, a 2022 study provides reassurance regarding the safety of co-prescribing statins and SSRIs, citing potential synergistic benefits for patients with comorbid depression and cardiovascular conditions [46]. These findings highlight the complexity of SSRI interactions, emphasizing the necessity of tailored approaches to minimize risks while ensuring therapeutic efficacy across diverse patient profiles.

Evaluation and management of similar cases

The management of SSRI-induced bradycardia follows the general principles of evaluating and treating symptomatic bradycardia. This begins with a thorough history and focused physical examination to identify potential causes of sinus node dysfunction. It is crucial to distinguish between intrinsic factors, such as primary cardiac abnormalities, and extrinsic factors, including other medications such as beta-blockers and non-dihydropyridine (non-DHP) calcium channel blockers or systemic conditions, e.g., hypothyroidism that may contribute to bradycardia, usually by impairing the sinus node or causing AV nodal block [47]. Given that SSRI-induced bradycardia can present with syncope, other potential causes of syncope, such as vasovagal or orthostatic etiologies, must be ruled out [47,48].

Furthermore, exploring the context of symptom onset helps in pinpointing precipitating factors, such as nocturnal bradycardia, which may suggest obstructive sleep apnea (OSA). Additionally, a meticulous medication history is essential to establish any temporal relationship between the use of an SSRI and the onset of bradycardia [17,47]. During the physical examination, clinical signs such as low pulse rate, decreased heart rate, or peripheral pulse deficits should be assessed to confirm the diagnosis [47].

SSRI-induced bradycardia may be detected during routine follow-ups in the clinic; however, some patients may present to the ER with related symptoms. Confirmation of bradycardia requires electrocardiography, with a standard 12-lead ECG being the primary diagnostic tool to assess the cardiac condition. For patients experiencing intermittent symptoms, ambulatory ECG monitoring, such as Holter monitoring, may be necessary [47]. To evaluate reversible causes and overall cardiac function, additional investigations such as a basic metabolic panel, cardiac enzymes, complete blood count, thyroid function tests, and electrolyte, urea, and creatinine levels are recommended. Echocardiography may also be utilized to assess structural and functional cardiac abnormalities, with further ancillary tests aiding in identifying contributing factors [17,47,49]. Certain clinicians may take an additional step to determine a causal link between a drug and the resulting adverse reaction by utilizing the Adverse Drug Reaction Probability Scale, also known as the Naranjo Algorithm. While this tool was created for use in controlled trials and the evaluation of new medications, rather than routine clinical settings, it remains straightforward to use and is widely applied [17,50,51].

Management of SSRI-induced bradycardia is based on symptom severity. Asymptomatic patients typically do not require intervention. For symptomatic cases, discontinuation of the SSRI or dose reduction to the minimum effective level may resolve symptoms. If alternatives are unavailable, other options, such as pacemaker implantation, may be considered while maintaining the current medication. Once stabilized, patients should be closely monitored via telemetry, and a cardiology consultation should be obtained for further evaluation, follow-up, and comprehensive management to ensure optimal care [47,52].

Aging and SSRI-induced bradycardia**

Sertraline and other SSRIs are commonly used to manage mood disorders, including anxiety and depressive disorders. Studies have reported extensive variability in the pharmacokinetics of these medications in adults [53]. A study conducted in a Chinese population found that aging was a significant factor in the clearance of sertraline, with patients exhibiting decreased clearance and increased exposure to the drug with increasing age [54]. This increased drug concentration may contribute to a higher likelihood of side effects, including bradycardia, in older patients.

A study by Jin et al. demonstrated that patients younger than 30 years cleared escitalopram faster than patients aged 30-50 years and those older than 50 years. Slower activity of CYP2C19, a phase 1 enzyme, contributed to this decreased drug clearance, highlighting the impact of pharmacokinetics on drug adverse effects and toxicity [55].

While opinions are divided on the impact of aging on other aspects of pharmacokinetics, such as absorption and distribution, aging has been shown to affect the phase 1 metabolism of several drugs, whereas phase 2 reactions remain largely unaffected [56]. Additionally, degenerative changes in the conducting system of the heart, commonly seen with aging, may contribute to the risk of bradycardia. Age-related conditions, such as sick sinus syndrome (SSS) or pre-existing sinus node dysfunction, may lead to symptomatic bradycardia in older patients or increase the likelihood of this condition with SSRI use [17]. The interplay between aging, drug clearance, autonomic nervous system changes, and medication use increases the risk of bradycardia in older adults. As such, healthcare providers must consider these factors when evaluating and managing bradycardia in this population. By doing so, they can provide personalized care and mitigate the risks associated with bradycardia.

## Conclusions

In summary, this case report highlights the challenge of managing bradycardia induced by SSRIs in a 63-year-old patient. Despite medication adjustments, the bradycardia persisted until bupropion was initiated, resolving the issue. The case underscores the importance of personalized care, close monitoring, and further research into SSRI-induced bradycardia. Tailored approaches, interdisciplinary collaboration, and consideration of individual risk factors are vital in optimizing patient outcomes in such scenarios.
